# The Association Between App-Administered Depression Assessments and Suicidal Ideation in User Comments: Retrospective Observational Study

**DOI:** 10.2196/18392

**Published:** 2020-08-04

**Authors:** Shelly DeForte, Yungui Huang, Tran Bourgeois, Syed-Amad Hussain, Simon Lin

**Affiliations:** 1 Abigail Wexner Research Institute Nationwide Children's Hospital Columbus, OH United States

**Keywords:** mobile health, mHealth, depression, qualitative research, mental health

## Abstract

**Background:**

Many people use apps to help understand and manage their depression symptoms. App-administered questionnaires for the symptoms of depression, such as the Patient Health Questionnaire-9, are easy to score and implement in an app, but may not be accompanied by essential resources and access needed to provide proper support and avoid potential harm.

**Objective:**

Our primary goal was to evaluate the differences in risks and helpfulness associated with using an app to self-diagnose depression, comparing assessment-only apps with multifeatured apps. We also investigated whether, what, and how additional app features may mitigate potential risks.

**Methods:**

In this retrospective observational study, we identified apps in the Google Play store that provided a depression assessment as a feature and had at least five user comments. We separated apps into two categories based on those having only a depression assessment versus those that offered additional supportive features. We conducted theoretical thematic analyses over the user reviews, with thematic coding indicating the helpfulness of the app, the presence of suicidal ideation, and how and why the apps were used. We compared the results across the two categories of apps and analyzed the differences using chi-square statistical tests.

**Results:**

We evaluated 6 apps; 3 provided only a depression assessment (assessment only), and 3 provided features in addition to self-assessment (multifeatured). User comments for assessment-only apps indicated significantly more suicidal ideation or self-harm (n=31, 9.4%) compared to comments for multifeatured apps (n=48, 2.3%; *X*^2^_1_=43.88, *P*<.001). Users of multifeatured apps were over three times more likely than assessment-only app users to comment in favor of the app’s helpfulness, likely due to features like mood tracking, journaling, and informational resources (n=56, 17% vs n=1223, 59% respectively; *X*^2^_1_=200.36, *P*<.001). The number of users under the age of 18 years was significantly higher among assessment-only app users (n=40, 12%) than multifeatured app users (n=9, 0.04%; *X*^2^_1_=189.09, *P*<.001).

**Conclusions:**

Apps that diagnose depression by self-assessment without context or other supportive features are more likely to be used by those under 18 years of age and more likely to be associated with increased user distress and potential harm. Depression self-assessments in apps should be implemented with caution and accompanied by evidence-based capabilities that establish proper context, increase self-empowerment, and encourage users to seek clinical diagnostics and outside help.

## Introduction

Digital devices have become an essential part of our lives. People increasingly rely on digital content and functionalities delivered through mobile apps for information, entertainment, daily task management, work functions, and even for health-related activities, such as symptom tracking, diagnosis, and digital health treatment. Increased patient willingness and appetite to adopt mental health apps and share data [1,2] has resulted in a growing market for mental health apps. Mental health apps could play a critical role in addressing unmet needs in mental health disease screening, self-management, monitoring, and health education [3,4]. This role is especially needed, considering the severe shortage of mental health professionals and the long wait for mental health services.

However, most publicly available mental health apps have not been scientifically validated [5-7], provide inaccurate information, and do not follow clinical guidelines [8]. Often these apps also employ misleading marketing tactics [9] that may suggest to the end user an unearned medical or algorithmic authority. App-administered questionnaires for the symptoms of depression, such as the Patient Health Questionnaire-9 (PHQ-9) [10], are of particular concern because the results are often presented without proper context or access to additional resources. Although safety concerns have been raised about leaving patients to their own devices [11,12], it has not been established whether apps that administer a depression self-assessment are beneficial or dangerous, especially when an app solely offers a depression self-assessment. Given that people with mental health concerns are at high risk, it is essential to evaluate the safety of readily available mobile apps and the presence or absence of mitigating factors to reduce this risk.

In this study, we assessed the self-reported user experience using user comments publicly posted for app review. This study aimed to compare user experiences with apps that are assessment-only for depression with no supportive content to those that have additional resources and contextual information for depression. We hypothesized that consumers using assessment-only apps might report more distress without proper support, while those using multifeatured apps might fare better with additional supportive information and contexts. Our objective was to determine to what extent and how app-administered depression self-assessments may be associated with a perceived benefit versus self-reported distress or harm.

## Methods

Due to the public availability of these comments, this study was exempt from the requirements for institutional review board approval.

### Inclusion Criteria

We limited our analysis to apps available in Google Play store, as we were unable to extract comments automatically from the Apple App store. This study also included only those apps that offered a depression assessment feature and had more than five user comments.

### Data Collection

Author SD searched the Google Play store on October 15, 2018, with Google Chrome in incognito mode using the term “depression test.” SD read each app description and selected apps that met the inclusion criteria. Author SAH manually visited each Google Play app web page on October 19, 2018, selected “Read All Reviews,” scrolled downward until the page had loaded all of the reviews, and then selected “view page source” from the Google Chrome browser menu. After saving these pages’ source codes as files, author SAH developed a custom web scraping script to remove extraneous information (eg, HTML coding, rating), extract the user comments from each file, and compile them for analysis. This script used the Python library BeautifulSoup 4 (PyPi) to walk through the source page’s HTML tree and then used regular expressions to extract the relevant information.

### Data Analysis

The apps were categorized as those that provide only a depression assessment (assessment-only) and those having other features in addition to the assessment (multifeatured).

This observational study employed the theoretical thematic analysis approach defined by Braun and Clarke [13] to analyze the qualitative data of user comments. Guided by our research interests to evaluate whether the apps may be associated with perceived benefit or harm, we utilized a hybrid inductive and deductive framework. We adopted preconceived themes of benefit and distress but induced other themes by reading and coding the user comments. We first familiarized ourselves with the data, highlighting relevant words, phrases, and sentences related to helpfulness, risks, and functionalities. We began with free-form thematic tagging before grouping based on similar themes. Then we generated new themes, reviewed and finalized the definitions of themes, and finally coded all comments using the definitions and guideline. Coding for all themes was defined after reviewing the data multiple times. The full guideline and results can be found in the Results section. SD performed the initial manual thematic coding and grouping, and TB retagged the comments blind using the thematic scheme developed by SD. The final tagging was obtained based on discussion and consensus for any discrepancies, and a third independent coder (SAH) settled any disagreements. The prevalence-adjusted and bias-adjusted κ (PABAK) score determined inter-rater reliability.

Pearson chi-squared test with Yates continuity correction was used to analyze the difference between the assessment-only apps and multifeatured apps. The degree of freedom of the chi-squared test is 1. The significance level was set at *P*<.05.

## Results

### Apps Included in the Study

The apps that met the inclusion criteria, number of comments retrieved for each app, and the number of times each app had been rated are listed in Table 1. While ratings were not factored into the thematic coding, the number of ratings might reflect the relative user base of each app, as no additional data was available to indicate the actual number of users. Eight apps with depression assessments were excluded due to having fewer than five comments.

**Table 1:** The apps included in this analysis.

Features	Google Play app ID	In-app purchase	Ratings, n^a^	Comments, n^b^
Multifeatured^c^ (proprietary assessment)^d^	de.moodpath.android	Yes	7062	1226
Multifeatured (PHQ-9^e^)	com.moodtools.moodtools	Yes	2871	788
Multifeatured (modified PHQ-9)	com.williamalexander.android.depressiontracker	No	267	63
Assessment only^f^ (PHQ-9)	nl.japps.android.depressiontest	No	1408	264
Assessment only (proprietary assessment)^d^	com.programming.advanced.depressiontest	No	436	43
Assessment only (PHQ-9)	com.moodtools.depressiontest	No	213	27

^a^The number of ratings for each app, some of which may not include a comment.

^b^The number of ratings that included a written comment.

^c^Apps that have other capabilities aside from a depression assessment.

^d^A nonstandard assessment.

^e^PHQ-9: Patient Health Questionnaire-9 (a 9-question survey to determine the severity of depression symptoms).

^f^Apps that have a depression assessment only.

### Themes

Two tags, which capture benefit and harm for all apps selected, tested our hypothesis that more users of assessment-only apps than multifeatured apps reported distress and suicidal ideation (see Methods). In familiarizing ourselves with the comments and generating new themes, we were surprised to find that some comments indicated the users were probably minors. As pediatric users might be more vulnerable and should be considered separately, we created a youth (vs adult) tag to capture whether the post was apt to be from someone who was under 18 years of age. Three themes (tracking, report, and library) emerged to capture the useful app features in multifeatured apps, and three tags (management, self-knowledge, and therapy) were generated to capture the stated utility of additional features, when available. The full thematic coding guideline and finalized themes are described in Table 2.

**Table 2:** Thematic coding guidelines and finalized themes.

Theme		Description	Coding guideline
Youth		To capture whether the user was likely a youth or adult	Comment specifically mentioned the user age was under 18, or self-reported as being a child or being young. Comment indicated that they still needed their parents’ help or approval (which suggests they were under 18 years of age).
**Perception of helpfulness and harmfulness**			
	Helpful	To indicate that the app was affirmatively beneficial to the user.	Marked “Y” if the user said the app is good (or similar) but also mentions a specific app feature, indicating that they are using the feature and think it is good. Marked “N” if the user only said the app is “good” or complements a design aspect. Marked “Y” if the comment used the words “helpful” or “useful” to describe the app (aside from this, most short comments were an “N.” Marked “Y” if the user stated that the app would be generically beneficial for depression, but mark “N” if the user also noted the app was not helpful for them. Marked “Y” if the app prompted them to get professional help. Marked “Y” if the user said they love the app or similar.
	Distress	To capture suicidal ideation or self-harm	Marked “Y” for any comments like “kill me now” or “I want to die” or “why am I alive?” Marked “Y” for comments where they talked about someone else who is suicidal. Marked “Y” for comments that talked about hospitalization for past suicide attempts. Marked “Y” for comments that indicated the app 'saved their life' or app is a “lifesaver.” Marked “N” if the comment only referenced the safety plan.
**Features mentioned (for multifeatured only)**			
	Tracking	To capture whether this app was used as a tracking tool	Comments mentioned “tracking” or used similar terminology. Mood tracking, journaling, and cognitive behavioral therapy were grouped under this tag. The key distinguisher here was activities over time. Since the Moodtools app calls journaling a “Thought Diary,” any mention of mood tracking, thought diary or journal features were included with this tag. In the Moodpath app, a series of questions tracked mood, so references to a “questionnaire” were tagged with tracking.
	Reporting	To capture any mention of the depression assessment or waiting for the depression assessment results.	For Moodtools, the users may mention a “report” or a “doctor's note.” Since the depression assessment was meant to be shared with the doctor, tagged comments with “Y” when a user mentioned sharing results with their doctor. For Moodtools, since the assessment follows two weeks of mood tracking, we also marked references to waiting for two weeks as references to the depression assessment, even without explicitly mentioning the evaluation. Marked “Y” any mentions of a “test,” “diagnosis,” “results,” or “accuracy,” which probably refer to the assessment
	Library	To encapsulate features that can be used once and be useful; are not ongoing tracking but are also not references to the depression assessment.	Terms like “resources,” “educational,” “informative,” etc, suggest that this tag was appropriate. This tag encompassed mentioning information, activities, videos, and the “Safety plan” in Moodtools.
**How and why it helps (for multifeatured only)**			
	Management	To capture using the app for managing depression, moods, or other mental illness through activities done over time with broader interpretation.	This tag overlapped with the “Tracking” tag frequently. The comment must indicate that the user is using the tracking feature to help their symptoms. “Great for tracking” would be an example of a comment that was tagged “Management” but not “Tracking.” Included comments that captured activities that would relieve symptoms of depression, such as “I can express myself,” “it's like talking to a friend,” “I like that it checks in on me.” Included comments in which the user expressed that the app helped them feel better. Included comments that referenced depression or other mental illness and indicated that they were using the app to manage this state. Included comments that indicated the app helped the user handle or manage things. Included comments that referenced self-knowledge over time for symptom management. Note: this tag overlapped with the self-knowledge tag.
	Self-knowledge	To capture comments that indicate that the user learned more about themselves through use of the app.	Despite some overlap with the “Library” tag, we were looking for more personal comments that indicated the user had learned something about themselves. This tag overlapped with the management tag when the user referenced that they have learned more about themselves and helped them to manage their symptoms. Tagged comments that referred to learning about patterns or learning new skills. Words like “insightful” or “educational” frequently indicated use for self-knowledge.
	Therapy	Infers if the user is in therapy, using the app with a therapist, or prompted by the app to go to a therapist.	Included any comments by therapists. Included comments that generically mention that the user could or might have used the app with a therapist or medical professional. Included comments such as “you should use this app with a professional” because it indicates that using the app with a therapist is a priority for the user.

### Coding Results

The PABAK scores for all tags, which were used to determine inter-rater reliability, 0-1 with 1.0 being the most reliable, were between 0.737 and 0.996 after the initial tagging (Multimedia Appendix 1), except for the “Help” tag for the multifeatured comments which had a PABAK score of 0.584. The two coders decided to treat comments that indicated general enthusiasm for the app as “helpful” comments, resolving the conceptual discrepancy that resulted in the low PABAK score with the updated PABAK score for Help of 0.682 and 95% CI 0.649-0.713.

The results of the thematic coding after a third coder adjudicated the final decisions on discordant coding are summarized in Table 3.

**Table 3:** Thematic coding results summary.

Theme	Assessment only (n=329), n (%)	Multifeatured (n=2069), n (%)	*P* value (assessment-only vs multifeatured)
Helpful	56 (17.02)	1223 (59.11)	<.001
Distress	31 (9.42)	47 (2.27)	<.001
Youth (vs adult)	40 (12.16)	9 (0.43)	<.001
Tracking	N/A^a^	509 (24.60)	N/A
Report	N/A	180 (8.70)	N/A
Library	N/A	253 (12.23)	N/A
Management	N/A	438 (21.17)	N/A
Self-knowledge	N/A	359 (17.35)	N/A
Therapy	N/A	118 (5.70)	N/A

^a^N/A: not applicable.

### Thematic Analysis

The most common words in all app comments were words with the root “help*” (1007 occurrences) and “depress*” (443 occurrences). Of the multifeatured app comments, 59% (1223/2069) expressly indicated the app was beneficial in some way, which is statistically different from 17% (56/329) of the comments for the assessment-only apps (*X*^2^_1_=200.36, *P*<.001). Many comments from assessment-only apps simply reported the score the user received or expressed powerlessness or frustration over their self-diagnosed condition. Of the comments for multifeatured apps, 2.3% (31/329) indicated suicidal ideation or self-harm, compared to 9.4% (47/2069) of the comments for assessment-only apps, representing a statistically significant 4-fold increase (*X*^2^_1_=43.88, *P*<.001).

Of users who self-reported their age category (youth vs adult), 12% (40/329) of comments from assessment-only app users indicated they were under 18 years of age, compared with 0.04% (9/2069) for the multifeatured app users, a statistically significant difference (*X*^2^_1_=189.09, *P*<.001). The *P* values of the comparison tests between depression test-only and multifeatured apps are listed in Table 3.

**Figure 1:**
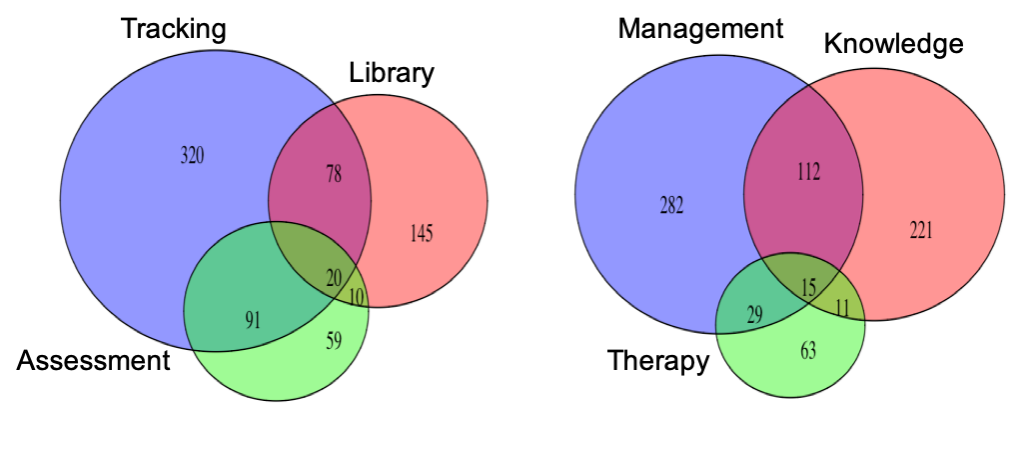
Comments that indicate app features being used (left) and explanations of app use and why it is beneficial (right). ‘Tracking’ refers to mood tracking or journal features, ‘Library’ refers to informational resources, videos, or prompts for activities, and ‘Assessment’ refers to the depression assessment (left). ‘Management’ refers to comments that indicate applying the app for self-management of moods or mental health over time, ‘Knowledge’ refers to self-knowledge or insight gained from using the app, while ‘Therapy’ refers to app use in conjunction with some kind of clinical therapy.

As indicated by the coding guideline, additional app features for multifeatured apps were grouped into tracking, library (informational resources), and depression assessment (report theme) categories. Comments mentioning tracking or informational resources (Library) appear 664/2069 times (32%) versus 180/2069 (9%) for the depression assessment. Users who discussed how and why they used the multifeatured apps most often referenced self-management of moods and using the apps to understand triggers and improve their state of mind (438/2069, 21%; Table 3). Others referenced how the apps allowed them to increase self-awareness and validate misunderstood feelings (359/2069, 17%). Few people mentioned using the app with a clinical provider (118/2069, 6%). The numbers of comments reflecting the induced themes for the multifeatured apps and the numbers of comments covering multiple themes are illustrated in Figure 1.

## Discussion

### Principal Findings

Our study supported our hypothesis that compared to users of multifeatured apps, assessment-only app users reported more distress, as seen in the significantly more substantial amount of comments indicating suicidal ideation. Additionally, users of assessment-only apps were more likely to be under 18 years of age than those using multifeatured apps. Compared to users of assessment-only apps, more users of multifeatured apps commented in favor of the apps’ helpfulness, mood tracking, journaling, and informational resources.

Our findings were consistent with other studies that showed that people who used health apps valued the ongoing tracking features greatly [14] and suggested that standalone apps that only administered a depression assessment were less beneficial than multifeatured apps. Alarmingly, we observed a remarkable 4-fold increase in comments indicating suicidal ideation and self-harm in the assessment-only apps, suggesting these apps may be more associated with potential harm. In another study, users scored higher on suicidal ideation indicators when the PHQ-9 was given via an app versus a clinician [15], indicating the importance of clinical guidance and resources at the time of assessment. While some evidence suggested that the privacy afforded by an app might increase self-disclosure [16-18], our analysis, which only compared apps, did not provide sufficient data to derive this conclusion for depression self-assessments. Instead, our results suggested that self-assessments given without proper support and context may be associated with triggering and exacerbating suicidal ideation. An underexplored factor in the literature is whether the self-diagnosis of depression through an app may induce *demoralization.* Demoralization is characterized by a sense of disheartenment and disempowerment that can follow a severe diagnosis and which is significantly associated with suicidal ideation [16]. We think this potentially confounding factor merits further study. Activities that lead to self-empowerment and self-awareness can decrease depressive symptoms [19,20], and an increase in perspective and perceived self-control can directly combat demoralization [21]. The additional features offered by the multifeatured apps might have provided the functionalities and engagement needed to enhance their self-awareness and sense of self-empowerment.

### Limitation and Challenges

A fundamental limitation of this observational study is that participants were self-selecting, in addition to the small sample size of the three assessment-only apps and the three multifeatured apps. The expressed opinions through user comments might not be representative of the user population due to the selection bias. Also noted in this comparative analysis is the highly disparate number of comments on the two types of apps. The smaller number of comments in the assessment-only apps might not be representative of the underlying user base. The retrospective nature of this research precluded obtaining user assessments of all the different themes we are trying to analyze from all the users. Instead, we had to rely solely on the publicly available comments in the app store. Inferring user opinion was also a challenge. Even though we followed a clearly defined coding guideline for consistency, coders still had different interpretations. We recognize that despite our best efforts, we may have misrepresented what users tried to convey.

This observational study is insufficient to establish causality. The assessment-only apps that we studied appear to have a higher number and proportion of underage users. This more substantial proportion might be due to the app’s simplicity and the likely associated ease of use, the free assessment, or possibly due to statistical variation resulting from a lack of a larger sample of data. Without a proper study design on a more extensive user base, we are unable to explain the reasons underlying the difference in the frequencies of underage users in the two types of apps. However, we feel that our results warrant caution and further study on the use and development of mental health assessment apps. Especially concerning is the large proportion of adolescent users self-reporting suicidal ideation in the user comments of these apps. With the rising pediatric suicide rate [22], adolescents need more protective interventions against potential harms. All the best intentions of app creators to fill in the resource gaps before first mental health visits or between visits, the desire to give patients independence and privacy, and the goodwill to offer cheaper alternatives to clinical appointments might not be enough to keep a good balance on benefits and risks when implementing mental health apps. This caution is particularly important for younger users.

Finally, the landscape of health apps is extraordinarily dynamic and rapidly evolving [23]. We were only able to capture the data at one point and unable to validate our findings against more current data from 2019 to 2020, which is another limitation of this study. Nonetheless, we are confident our research provides an important data point in the continuous timeline, and our conclusions are expected to stand with the newer data.

### Conclusions

In summary, we express our reservation about using mental health assessment-only apps without providing additional resources and functionalities. We also recommend that evidence-based, mitigating activities (eg, mood tracking, journaling, educational materials, and self-empowerment and self-awareness activities) should accompany any app that can lead to the self-diagnosis of depression or other mental health conditions. Assessment-only apps should firmly emphasize that assessment done in the app is not diagnostic, provide a clear recommendation to follow up with health professionals to conduct clinical diagnostic testing, and provide links to additional evidence-based information on depression [24,25]. If an assessment-only app can give the proper warnings and provide informational links, they can still be beneficial, especially to people who would want to an initial understanding of their mental health in private, or people with limited financial means. The advocated enhancement should be straightforward to implement and should not add much development costs to the apps. It might be more beneficial for the multifeatured app to offer the assessment module for free. These additions need to be balanced by engagement design factors [5] to avoid deterring use by younger users.

Even though multifeatured mental health apps seem to mitigate some risks of harm, additional larger-scale prospective research studies are needed to accurately assess the long-term benefits and risks of mental health apps [1,4]. All aspects of apps, including user experience, user engagement, age appropriateness of contents, values of different features, impacts attributed to apps, and others, should be investigated. We agree with many research studies that recommend apps incorporate evidence-based content [5], adopt a digital health app development standard [3,26], rely on clinician recommendations of validated apps versus social media, personal searches, or word-of-mouth to explore or adopt a health app [27,28] and require app stores to standardize reporting. We realize that implementation of these recommendations presents significant challenges requiring collaboration and development efforts across multiple fields, including clinical research, mobile health research, health IT industry, regulation, clinical practice, and others. Nevertheless, these standardizations and safeguards are essential to help patients find validated apps, prevent harm, and assure mental health apps create the value they claim to provide.

## Appendix

Multimedia Appendix 1 Thematic coding interrater reliability.

## Abbreviations

PABAK: prevalence-adjusted and bias-adjusted κ
PHQ-9: Patient Health Questionnaire-9

## References

[1] BinDhim NF, Alanazi EM, Aljadhey H, Basyouni MH, Kowalski SR, Pont LG, Shaman AM, Trevena L, Alhawassi TM (2016). Does a Mobile Phone Depression-Screening App Motivate Mobile Phone Users With High Depressive Symptoms to Seek a Health Care Professional's Help?. J Med Internet Res.

[2] Di Matteo D, Fine A, Fotinos K, Rose J, Katzman M (2018). Patient Willingness to Consent to Mobile Phone Data Collection for Mental Health Apps: Structured Questionnaire. JMIR Ment Health.

[3] BinDhim NF, Shaman AM, Trevena L, Basyouni MH, Pont LG, Alhawassi TM (2015). Depression screening via a smartphone app: cross-country user characteristics and feasibility. J Am Med Inform Assoc.

[4] Batra S, Baker RA, Wang T, Forma F, DiBiasi F, Peters-Strickland T (2017). Digital health technology for use in patients with serious mental illness: a systematic review of the literature. Med Devices (Auckl).

[5] Stawarz K, Preist C, Tallon D, Wiles N, Coyle D (2018). User Experience of Cognitive Behavioral Therapy Apps for Depression: An Analysis of App Functionality and User Reviews. J Med Internet Res.

[6] Wasil AR, Venturo-Conerly KE, Shingleton RM, Weisz JR (2019). A review of popular smartphone apps for depression and anxiety: Assessing the inclusion of evidence-based content. Behav Res Ther.

[7] Anthes E (2016). Mental health: There's an app for that. Nature.

[8] Martinengo L, Van Galen L, Lum E, Kowalski M, Subramaniam M, Car J (2019). Suicide prevention and depression apps' suicide risk assessment and management: a systematic assessment of adherence to clinical guidelines. BMC Med.

[9] Parker L, Bero L, Gillies D, Raven M, Mintzes B, Jureidini J, Grundy Q (2018). Mental Health Messages in Prominent Mental Health Apps. Ann Fam Med.

[10] Kroenke K, Spitzer RL, Williams JBW (2001). The PHQ-9. J Gen Intern Med.

[11] Ho Anita, Quick Oliver (2018). Leaving patients to their own devices? Smart technology, safety and therapeutic relationships. BMC Med Ethics.

[12] Lupton D, Jutel A (2015). 'It's like having a physician in your pocket!' A critical analysis of self-diagnosis smartphone apps. Soc Sci Med.

[13] Braun V, Clarke V, Rance N (2017). How to use thematic analysis with interview data Internet. The Counselling and Psychotherapy Research Handbook.

[14] Rubanovich CK, Mohr DC, Schueller SM (2017). Health App Use Among Individuals With Symptoms of Depression and Anxiety: A Survey Study With Thematic Coding. JMIR Ment Health.

[15] Torous J, Staples P, Shanahan M, Lin C, Peck P, Keshavan M, Onnela J (2015). Utilizing a Personal Smartphone Custom App to Assess the Patient Health Questionnaire-9 (PHQ-9) Depressive Symptoms in Patients With Major Depressive Disorder. JMIR Ment Health.

[16] Vehling S, Kissane DW, Lo C, Glaesmer H, Hartung TJ, Rodin G, Mehnert A (2017). The association of demoralization with mental disorders and suicidal ideation in patients with cancer. Cancer.

[17] Scott MA, Wilcox HC, Schonfeld IS, Davies M, Hicks RC, Turner JB, Shaffer D (2009). School-based screening to identify at-risk students not already known to school professionals: the Columbia suicide screen. Am J Public Health.

[18] Bradford S, Rickwood D (2015). Acceptability and utility of an electronic psychosocial assessment (myAssessment) to increase self-disclosure in youth mental healthcare: a quasi-experimental study. BMC Psychiatry.

[19] Kauer SD, Reid SC, Crooke AHD, Khor A, Hearps SJC, Jorm AF, Sanci L, Patton G (2012). Self-monitoring using mobile phones in the early stages of adolescent depression: randomized controlled trial. J Med Internet Res.

[20] Bastiaansen JA, Meurs M, Stelwagen R, Wunderink L, Schoevers RA, Wichers M, Oldehinkel AJ (2018). Self-monitoring and personalized feedback based on the experiencing sampling method as a tool to boost depression treatment: a protocol of a pragmatic randomized controlled trial (ZELF-i). BMC Psychiatry.

[21] Boscaglia N, Clarke DM (2007). Sense of coherence as a protective factor for demoralisation in women with a recent diagnosis of gynaecological cancer. Psychooncology.

[22] Segu S, Tataria R (2016). Paediatric suicidal burns: A growing concern. Burns.

[23] Olff M (2015). Mobile mental health: a challenging research agenda. Eur J Psychotraumatol.

[24] Hsin H, Torous J (2018). Creating boundaries to empower digital health technology. BJPsych Open.

[25] Silow-Carroll S, Smith B (2013). Clinical management apps: creating partnerships between providers and patients. Issue Brief (Commonw Fund).

[26] Van Velthoven MH, Smith J, Wells G, Brindley D (2018). Digital health app development standards: a systematic review protocol. BMJ Open.

[27] Schueller SM, Neary M, O'Loughlin K, Adkins EC (2018). Discovery of and Interest in Health Apps Among Those With Mental Health Needs: Survey and Focus Group Study. J Med Internet Res.

[28] Shen N, Levitan M, Johnson A, Bender JL, Hamilton-Page M, Jadad AAR, Wiljer D (2015). Finding a depression app: a review and content analysis of the depression app marketplace. JMIR Mhealth Uhealth.

