# Supplementary material for: The Association Between App-Administered Depression Assessments and Suicidal Ideation in User Comments: Retrospective Observational Study
Source: JMIR Mhealth Uhealth. 2020 Aug 4;8(8):e18392. doi: 10.2196/18392 (PMC7435620; doi:10.2196/18392)
Supplement: Multimedia Appendix 1 [file mhealth_v8i8e18392_app1.docx]

**Table A2. Thematic Coding Inter-Rater Reliability**

| Tag | Applied to | PABAK^1^ | Lower bound | Upper bound |
| --- | --- | --- | --- | --- |
| Helps | assessment-only | 0.793 | 0.717 | 0.855 |
|  | multi-featured | 0.584 | 0.548 | 0.620 |
| Distress | assessment-only | 0.884 | 0.822 | 0.93 |
|  | multi-featured | 0.967 | 0.954 | 0.977 |
| Age < 18 | assessment-only | 0.933 | 0.881 | 0.966 |
|  | multi-featured | 0.996 | 0.990 | 0.999 |
| Tracking | multi-featured | 0.788 | 0.760 | 0.814 |
| Library | multi-featured | 0.906 | 0.886 | 0.924 |
| Assessment | multi-featured | 0.790 | 0.762 | 0.816 |
| Management | multi-featured | 0.754 | 0.724 | 0.781 |
| Knowledge | multi-featured | 0.737 | 0.706 | 0.766 |
| Therapy | multi-featured | 0.950 | 0.934 | 0.962 |

**^1^ Prevalence-adjusted and bias-adjusted kappa results based on two coders.**
